# Exosome from IFN-γ-Primed Induced Pluripotent Stem Cell-Derived Mesenchymal Stem Cells Improved Skin Inflammation and Barrier Function

**DOI:** 10.3390/ijms241411635

**Published:** 2023-07-19

**Authors:** Jin Yoon, Seul Ki Lee, Arum Park, Jiho Lee, Inuk Jung, Kun Baek Song, Eom Ji Choi, Soo Kim, Jinho Yu

**Affiliations:** 1 Asan Institute for Life Sciences, Asan Medical Center, Seoul 05505, Republic of Korea; yoonjin8787@gmail.com (J.Y.); alal6824@gmail.com (A.P.); gis6093@daum.net (J.L.); 2 Brexogen Research Center, Brexogen Inc., Seoul 05855, Republic of Korea; seulki.lee@brexogen.com; 3 School of Computer Science and Engineering, Kyungpook National University, Daegu 41566, Republic of Korea; inukjung@knu.ac.kr; 4 Department of Pediatrics, Asan Medical Center, University of Ulsan College of Medicine, Seoul 05505, Republic of Korea; dizz2@hanmail.net (K.B.S.); calleomza@naver.com (E.J.C.)

**Keywords:** atopic dermatitis, exosome, interferon-gamma, mesenchymal stem cell

## Abstract

The pathogenesis of atopic dermatitis (AD) is multifactorial, including immune dysregulation and epidermal barrier defects, and a novel therapeutic modality that can simultaneously target multiple pathways is needed. We investigated the therapeutic effects of exosomes (IFN-γ-iExo) secreted from IFN-γ-primed induced pluripotent stem cell-derived mesenchymal stem cells (iMSC) in mice with *Aspergillus fumigatus*-induced AD. IFN-γ-iExo was epicutaneously administered to mice with AD-like skin lesions. The effects of IFN-γ-iExo treatment were investigated through clinical scores, transepidermal water loss (TEWL) measurements, and histopathology. To elucidate the therapeutic mechanism, we used an in vitro model of human keratinocyte HaCaT cells stimulated with IL-4 and IL-13 and performed extensive bioinformatics analysis of skin mRNA from mice. The expression of indoleamine 2,3-dioxygenase was higher in IFN-γ primed iMSCs than in iMSCs. In human keratinocyte HaCaT cells, treatment with IFN-γ-iExo led to decreases in the mRNA expression of thymic stromal lymphopoietin, IL-25, and IL-33 and increases in keratin 1, keratin 10, desmoglein 1, and ceramide synthase 3. IFN-γ-iExo treatment significantly improved clinical and histological outcomes in AD mice, including clinical scores, TEWL, inflammatory cell infiltration, and epidermal thickness. Bioinformatics analysis of skin mRNA from AD mice showed that IFN-γ-iExo treatment is predominantly involved in skin barrier function and T cell immune response. Treatment with IFN-γ-iExo improved the clinical and histological outcomes of AD mice, which were likely mediated by restoring proper skin barrier function and suppressing T cell-mediated immune response.

## 1. Introduction

Atopic dermatitis (AD) is a chronic and relapsing inflammatory skin disease characterized by pruritic skin lesions [1]. The pathogenesis of AD is typically defined by increases in T helper (Th) 2-mediated inflammatory responses, including the release of immunoglobulin E (IgE); recruitment of eosinophils; production of interleukin (IL)-4, IL-5, and IL-13; and Th1-dominant skin inflammation in the chronic phase [2,3,4,5]. Studies have also indicated that inflammation is initiated by skin barrier defects [1]. While the primary modes of treatment for AD are systemic and topical anti-inflammatory and immunosuppressive agents [6], biologics that block single pathways involved in the pathophysiology of AD have been recently introduced [7]. However, as AD is a multifactorial disease involving complex immunologic pathways, a new therapeutic modality that targets multiple pathways of immune regulation and the skin barrier is needed to effectively treat AD.

Mesenchymal stem cells (MSCs) are multipotent non-hematopoietic stem cells that can differentiate into various cell types [8,9,10] and elicit regenerative and anti-inflammatory effects [11]. Recent preclinical and clinical studies have demonstrated that administration of umbilical cord blood-derived MSCs reduced the severity of AD [12,13,14]. Human umbilical cord blood-derived MSCs effectively treat murine atopic dermatitis by activating NOD2, inhibiting mast cell degranulation [12]. Over a period of 3 months, approximately 55% of patients with AD demonstrated symptom improvement after receiving a high dosage of umbilical cord blood-derived MSCs [13]. Interferon-gamma (IFN-γ) is a pro-inflammatory cytokine involved in transcriptional regulation of immunologically relevant genes, and we reported that priming with IFN-γ enhanced the therapeutic effects of Wharton’s jelly-derived MSCs in a murine AD model and demonstrated the involvement of immune pathways in Wharton’s jelly-derived MSCs primed with IFN-γ by bioinformatics analyses [14].

Despite the increasing clinical applicability of MSCs, several limitations remain to be resolved. For example, the current protocol for isolation and culturing MSCs typically yields a heterogeneous population of cells [15], primarily attributed to the unavailability of definitive surface markers for MSCs during isolation. Additionally, MSCs have a limited potential to grow for an extended period and can easily become senescent [16,17]. Moreover, their biological characteristics, such as differentiation potential and immunomodulatory function, can be lost over several passages of culture [18].

Exosomes are cell-derived nano-sized particles that are produced by almost all cell types [19,20], and recent studies have shown that exosomes can carry or embed various active biological compounds by functioning as internal or membrane cargo [21,22,23]. Furthermore, studies have shown that the content of exosomes can be tailored by priming MSCs with physiological cues, thereby optimizing the therapeutic roles of exosomes [24]. Previous studies have demonstrated that exosomes are responsible for the therapeutic effects of MSC in various preclinical models of inflammatory and degenerative diseases [20,24,25], suggesting that they could become novel bioactive products that can be alternatively used to overcome the problems that arise in the use of MSCs. However, obtaining a sufficient quantity of parental cells to collect exosomes remains a major challenge due to replicative senescence and decreased biological function of MSCs during extended culture.

In light of these challenges, MSCs differentiated from induced pluripotent stem cells (iPSCs) [26] have the potential to become an ideal source of generating exosomes in large quantities [11,12]. iPSC-derived MSCs (iMSCs) can be easily obtained from iPSCs, which have an infinite ability to grow while maintaining the multi-lineage differentiation potential [11]. Although recent studies have shown that iMSCs might have distinct characteristics from MSCs, iMSCs do harbor MSC-like cellular characteristics such as cell surface-antigen expression profile and tri-lineage differentiation potential [13].

Here, we ©nvestigated the therapeutic effects of exosomes secreted from IFN-γ-primed iMSCs in mice with *Aspergillus fumigatus* (Af)-induced AD. We also investigated how these exosomes affect the immune response and skin barrier function.

## 2. Results

### 2.1. Characterization of IFN-γ-Primed iMSCs

**Figure 1:**
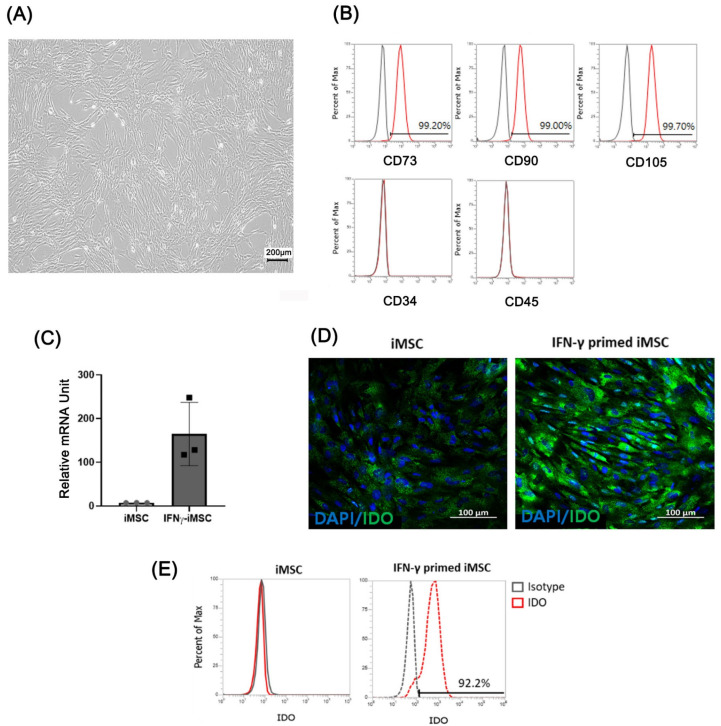
Characterization of IFN-γ-primed iPSC-derived MSCs (iMSCs) and verification of indoleamine 2,3-dioxygenase (IDO) expression. (**A**) Light microscopic image analysis of the morphology of IFN-γ-primed iMSCs. (**B**) Flow cytometry analysis of IFN-γ-primed iMSCs for surface markers related to exosomes (CD73, CD90, and CD105) and those not related to exosomes (CD34 and CD45). (**C**–**E**) mRNA and protein levels of IDO induced by IFN-γ treatment in iMSCs confirmed by quantitative reverse transcription-PCR (qRT-PCR) (**C**), immunocytochemistry (**D**), and flow cytometry©).

We investigated whether exosomes secreted from IFN-γ-primed iMSCs could cause immune suppression and skin barrier regeneration of induced AD under in vitro and in vivo conditions. We differentiated iPSCs from MSCs as previously described [27], and iMSCs were primed with IFN-γ for 24 h at 20 ng/mL to produce exosomes specialized in AD. Throughout the study, we included three different batch-produced IFN-γ-primed iMSCs to compare their characteristics and functionality. IFN-γ-primed iMSCs showed typical MSC-like morphology (Figure 1A), and flow cytometric analysis showed that iMSCs were positive for typical MSC surface markers such as CD73, CD90, and CD105 and negative for CD34 and CD45 (Figure 1B). After treating the iMSCs with IFN-γ, the mRNA and protein levels of IDO were measured through qRT-PCR, immunofluorescence, and FACS. The expression of IDO was higher in IFN-γ primed iMSCs than in iMSCs (Figure 1C–E).

### 2.2. Characterization of IFN-γ-iExo

**Figure 2:**
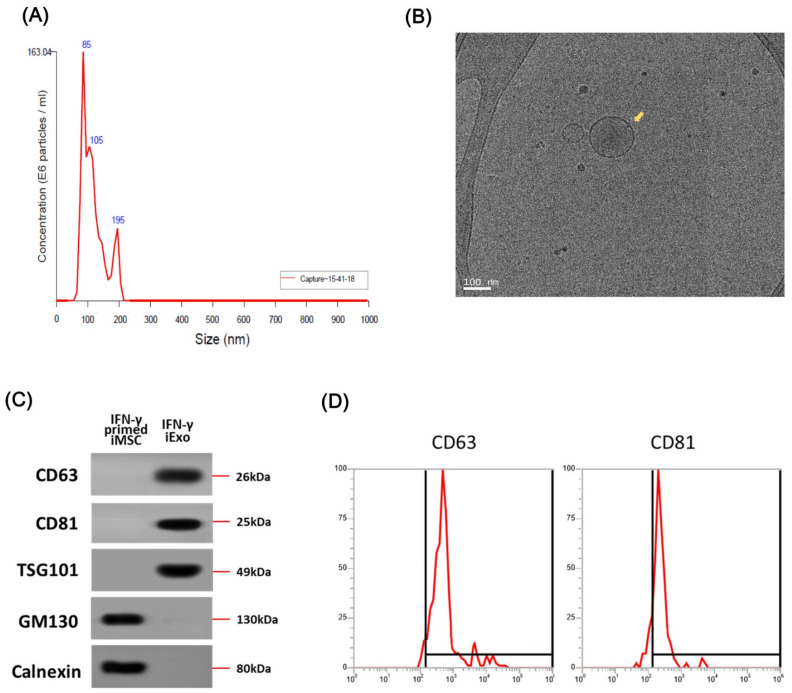
Characterization of exosomes produced from IFN-γ primed iMSCs (IFN-γ-iExo). (**A**) Size measurement of IFN-γ-iExo using nanoparticle tracking analysis (NTA). (**B**) Cryo-transmission electron microscopy (cryo-TEM) images of IFN-γ-iExo showing lipid bilayers and vesicular internal structures. (**C**) Immunoblotting analysis for exosome-related markers (CD63, CD81, and TSG101) and cell-specific markers (GM130 and Calnexin) in IFN-γ-iExo and their parental IFN-γ-primed iMSCs. (**D**) Flow cytometry analysis of CD63 and CD81 in IFN-γ-iExo. Y-axis represents the maximum percentage (%).

We collected IFN-γ-iExo from the supernatant of IFN-γ-primed iMSC culture by ultracentrifugation per the previously described protocol [21,28]. The average size of IFN-γ-iExo was 114 nm (range, 80–120) as indicated in nanoparticle tracking analysis (NTA) analyses (Figure 2A), which is typical of MSC-derived exosomes. Figure 2B shows the morphology of IFN-γ-iExo as visualized under transmission electron microscopy (TEM). Immunoblot analysis showed that IFN-γ-iExo expressed exosome-specific markers (CD63, CD81, and TSG101) and did not express negative markers for exosomes (GM130 and calnexin) (Figure 2C). Flow cytometry analysis showed that IFN-γ-iExo expressed exosome-specific markers CD63 and CD81 (Figure 2D).

### 2.3. Effect of IFN-γ-iExo on IL-4- and IL-13-Stimulated Human Keratinocyte HaCaT Cells

**Figure 3:**
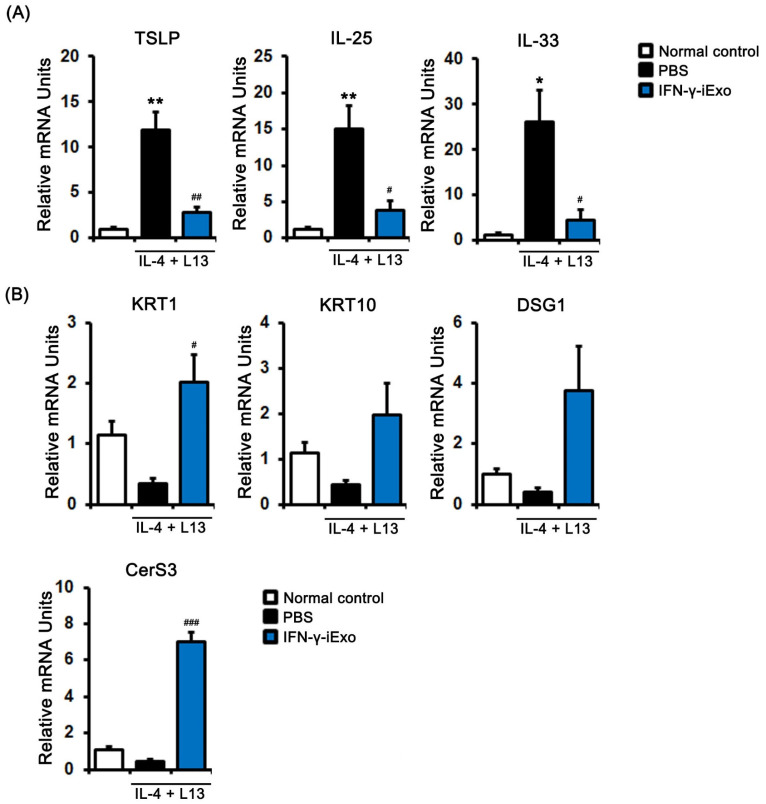
Effect of IFN-γ-iExo on Th2-stimulated human keratinocytes. HaCaT cells were co-treated with human recombinant IL-4 and IL-13 (both at 50 ng/mL) for 24 h, and subsequently incubated with IFN-γ-iExo (50 μg/mL) for 24 h. After treatment, the expression of immune response-related genes (TLSP, IL-25, and IL-33) (**A**) and genes involved in skin regeneration (KRT1, KRT10, DSG1, and CerS3) (**B**) were analyzed by qRT-PCR. Data are expressed as mean ± standard error of the mean. * *p* < 0.05, ** *p* < 0.001 vs. normal control ^#^ *p* < 0.05, ^##^ *p* < 0.01, ^###^ *p* < 0.001 vs. PBS treatment.

To investigate the effect of IFN-γ-iExo on the gene expression of human keratinocyte HaCaT cells stimulated with IL-4 and IL-13, we evaluated the expression of immune response-related genes. TSLP, IL-25, and IL-33 were significantly increased in human keratinocyte HaCaT cells stimulated with 50 ng/mL of IL-4 and IL-13, which was significantly decreased after treatment with IFN-γ-iExo (Figure 3A). We also investigated the effect IFN-γ-iExo on skin regeneration-related gene expression in human keratinocyte HaCaT cells stimulated with IL-4 and IL-13. We found that IFN-γ-iExo led to an increase in the mRNA expression levels of KRT1, KRT10, DSG1, and CerS3 (Figure 3B).

### 2.4. Therapeutic Effects of IFN-γ-iExo According to the Administration Route in the Af-Induced AD Mouse Model

**Figure 4:**
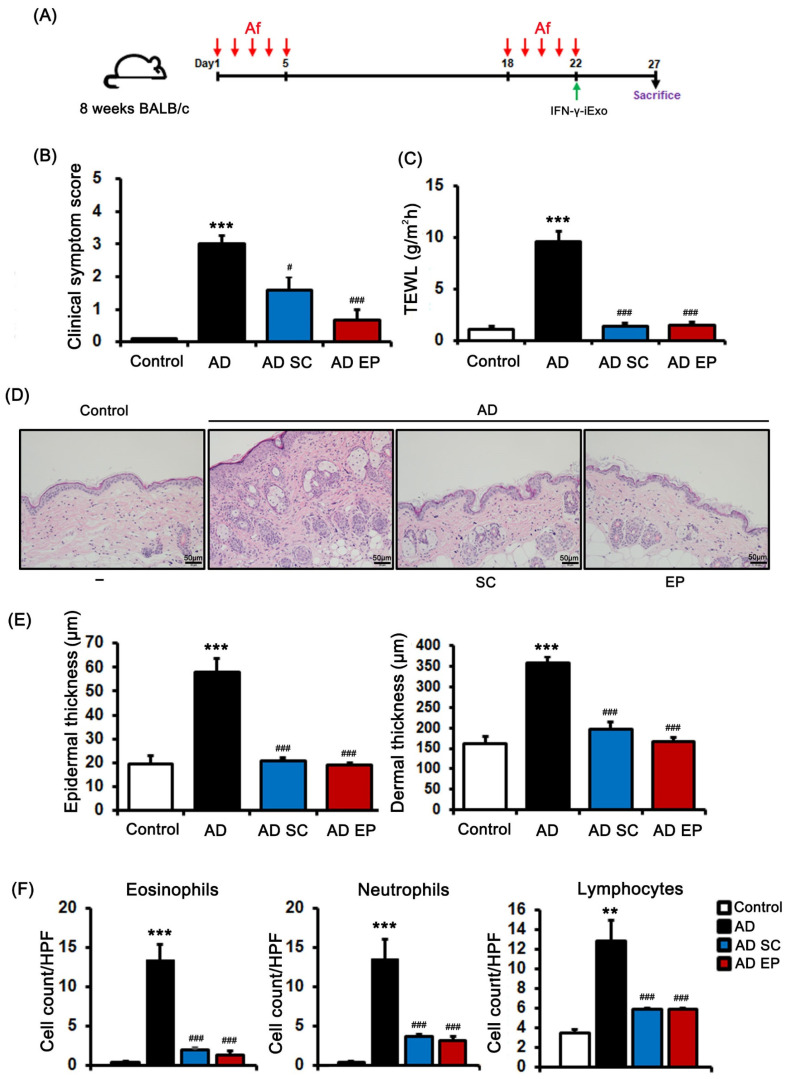
Therapeutic effects of IFN-γ-iExo according to administration route in Af-induced atopic dermatitis (AD) mouse model. (**A**) Schematic of the protocol used in this study. *Aspergillus fumigatus* (Af) extract (40 µg) was epicutaneously (EP) applied to the dorsal skin of the mice for 5 consecutive days, followed by another 5-day series of applications with an interval of 13 days. IFN-γ-iExo was administered either subcutaneously (SC) or EP on the last day of the second series of Af applications. The mice were divided into four groups: control, AD, AD treated with IFN-γ-iExo SC (AD SC), and AD treated with IFN-γ-iExo EP (AD EP). (**B**) Clinical symptom scores and (**C**) transepidermal water loss (TEWL) levels. (**D**) Hematoxylin and eosin (H&E) staining of the dorsal skin lesion. (**E**) Epidermal and dermal thickness of the dorsal skin lesions. (**F**) The numbers of eosinophils, neutrophils, and lymphocytes in the dorsal skin lesions. Data are expressed as mean ± standard error of the mean. ** *p* < 0.01, *** *p* < 0.001 vs. control; ^#^ *p* < 0.05, ^###^ *p* < 0.001 vs. AD group.

We evaluated the therapeutic effect of IFN-γ-iExo on AD induced by Af and then compared the effect of subcutaneous (SC) and epicutaneous (EP) administration of IFN-γ-iExo (Figure 4A). The AD group had significantly higher clinical symptom scores and TEWL than the control group (Figure 4B,C). However, clinical symptom scores and TEWL were significantly decreased in IFN-γ-iExo treatment groups regardless of the route of administration compared with the AD control group (Figure 4B,C). The lesional skin of the AD control group showed significantly increased epidermal thickness (57.9 ± 5.8 μm) and dermal thickness (358.1 ± 14.9 μm) (Figure 4D,E) than the control group (19.6 ± 3.4 μm, 161.3 ± 18.3 μm, respectively), as well as significantly higher numbers of eosinophils, neutrophils, and lymphocytes compared to those of the control mice (Figure 4D,F). On the contrary, both SC and EP administration of IFN-γ-iExo resulted in significantly lower epidermal (20.9 ± 1.2 μm, 19.1 ± 0.8 μm, respectively) and dermal thickness (195.4 ± 17.6 μm, 166.5 ± 11.8 μm, respectively) compared to the AD control group (Figure 4D,E), as well as a significant reduction in the number of eosinophils, neutrophils, and lymphocytes (Figure 4D,F). The therapeutic effect of IFN-γ-iExo was not significantly different between administration routes of SC and EP in the mouse model of Af-induced AD.

### 2.5. Dose-Dependent Effects of IFN-γ-iExo in the Af-Induced AD Mouse Model

**Figure 5:**
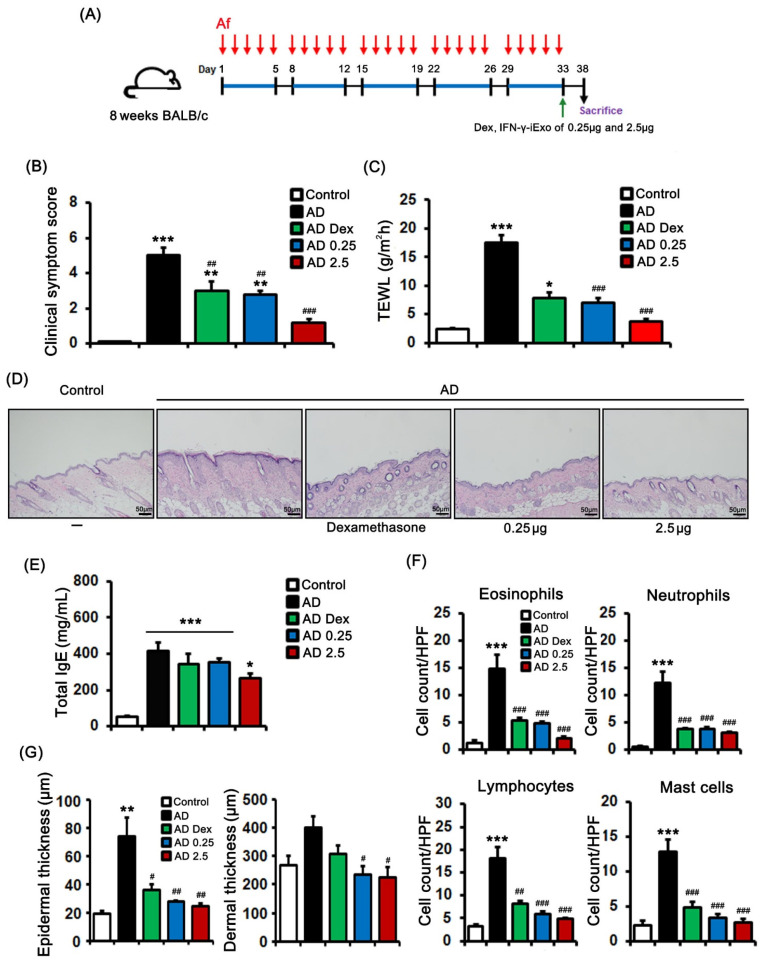
Dose-dependent effects of IFN-γ-iExo in *Aspergillus fumigatus* (Af)-induced atopic dermatitis mouse model. (**A**) Schematic of the schedule for animal experiments. *Aspergillus fumigatus* (Af) extract (40 µg) was epicutaneously applied to the dorsal skin of the mice for five consecutive days per week for 5 weeks. Dexamethasone 0.1%, low dose IFN-γ-iExo (0.25 μg), or high dose IFN-γ-iExo (2.5 μg) was administered epicutaneously on the last day of the second series of Af applications. The mice were divided into five groups: control, AD, AD treated with 0.1% dexamethasone (AD Dex), AD treated with 0.25 μg of IFN-γ-iExo (AD 0.25), and AD treated with 2.5 μg of IFN-γ-iExo (AD 2.5). (**B**) Clinical symptom scores and (**C**) transepidermal water loss (TEWL) levels. (**D**) Hematoxylin and eosin staining of the dorsal skin lesions. (**E**) Serum total IgE. (**F**) The numbers of eosinophils, neutrophils, lymphocytes, and mast cells in the dorsal skin lesions. (**G**) Epidermal and dermal thickness of the dorsal skin lesion. Data are expressed as mean ± standard error of the mean. * *p* < 0.05, ** *p* < 0.01, *** *p* < 0.001 vs. control; ^#^ *p* < 0.05, ^##^ *p* < 0.01, ^###^ *p* < 0.001 vs. AD-PBS group.

We evaluated the dose-dependent effect of EP-administered IFN-γ-iExo on AD (Figure 5A). Both 0.25 µg and 2.5 µg of IFN-γ-iExo, as well as dexamethasone, significantly improved clinical symptom scores and TEWL (Figure 5B,C). In terms of the dose-dependent effect of IFN-γ-iExo, while the low dose IFN-γ-iExo group and the dexamethasone groups had significantly worse clinical symptom scores and TEWL than did the control group, the high dose IFN-γ-iExo group showed comparable clinical scores and TEWL to the control group (Figure 5B,C). Furthermore, epidermal thickness was lower in mice administered dexamethasone (35.7 ± 3.9 μm) and 0.25µg (27.8 ± 0.8 μm) and 2.5 µg (24.3 ± 2.5 μm) of IFN-γ-iExo than in the AD control group (73.6 ± 14.0 μm) (Figure 5D,G). Consistently, dermal thickness was significantly lower in mice administered IFN-γ-iExo (low dose: 235.4 ± 27.7 μm, high dose: 224.9 ± 35.1 μm) than in the AD control group (400.3 ± 40.5 μm) (Figure 5D,G). In addition, both low and high doses of IFN-γ-iExo, as well as dexamethasone, significantly improved immune cell infiltration compared to the AD control group (Figure 5D,F). The total IgE levels in the serum of Af-treated mice were significantly higher than in the control group (Figure 5E), and treatment with 2.5 µg of IFN-γ-iExo showed a tendency to decrease the level of IgE compared to the AD control group (Figure 5F).

### 2.6. Top 15 Pathways Commonly Enriched in the Skin of the AD Mice Treated with IFN-γ-iExo

**Figure 6:**
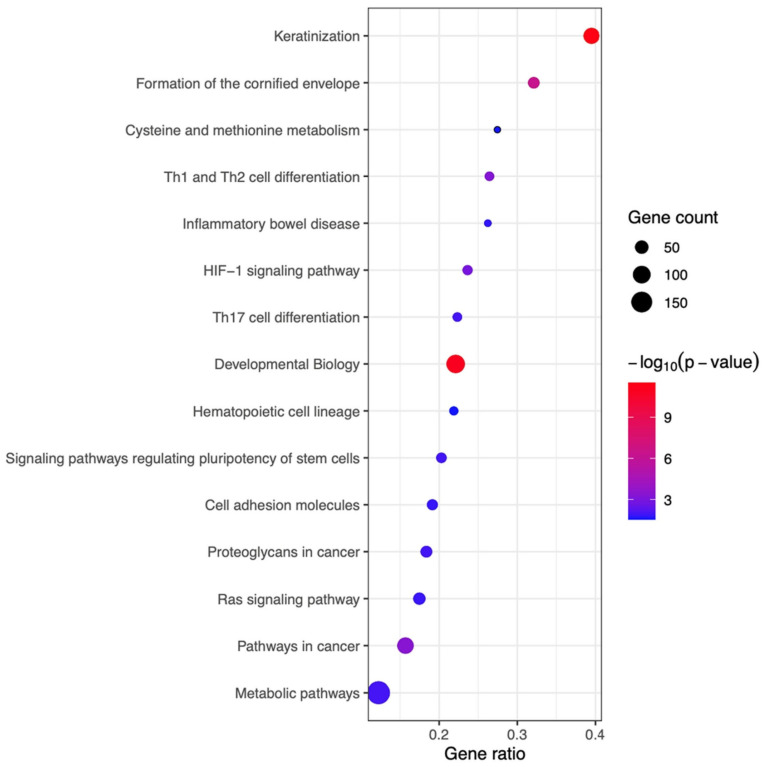
The Kyoto Encyclopedia of Genes and Genomes (KEGG) pathway enrichment analysis of differentially expressed genes (DEGs) in skin tissues obtained from AD mice treated with IFN-γ-iExo and those treated with PBS. Pathways with significantly enriched genes are shown in descending order of the gene ratio. The gene ratio refers to the ratio of DEGs in each pathway. The absolute number of DEGs in each pathway is represented by the size and the significance of the color of the dots. The color codes are shown in -log10 (*p*-value) form.

To investigate the mechanisms underlying the therapeutic effects of IFN-γ-iExo, we performed bioinformatics analyses of the skin mRNA (Figure S1). A total of 1992 differentially expressed genes (DEGs) were found in the skin of the AD mouse model according to treatment with IFN-γ-iExo. In bioinformatics analysis, a total of 15 significant pathways (*p*-value < 0.05) were identified (Figure 6). Canonical pathways in the skin of mice treated with IFN-γ-iExo were predominantly involved in skin barrier function (e.g., keratinization, formation of the cornified envelope) and T cell immune response (e.g., Th1, Th2, and Th17 cell differentiation).

## 3. Discussion

We have previously demonstrated that IFN-γ-primed WJ-MSCs improved AD-like skin lesions by immunomodulation in an Af-induced AD mouse model [14]. Here, we report that exosomes secreted by IFN-γ-iMSCs have typical shapes, structures, and markers of exosomes. Furthermore, the expression of IDO in iMSCs significantly increased upon IFN-γ priming. In human keratinocytes, IFN-γ-iExo treatment nullified the increase in the expression of immune response-related genes and increased the expression of skin regeneration-related genes. IFN-γ-iExo ameliorated AD-like skin lesions regardless of the route of administration (i.e., EP and SC), and the therapeutic effect was dose-dependent in Af-induced AD mice models. Furthermore, bioinformatics analysis of the skin mRNA of AD mice showed that IFN-γ-iExo treatment was predominantly involved in skin barrier function, including keratinization and formation of the cornified envelope, and in the T cell immune response, including Th1, Th2, and Th17 cell differentiation.

IFN-γ is a well-known inducer of immune responses, and IFN-γ-treated MSCs exhibit immunosuppressive functions in vitro and in vivo [10,14,29]. Several studies reported that IFN-γ increases the expression of IDO within MSCs [10,27,30,31,32], suggesting that the effect of exosomes derived from IFN-γ-primed iMSCs may be mediated by increased IDO expression. Increased IDO and kynurenine levels stimulate the expansion and stimulation of myeloid-derived suppressor cells [33], thereby inhibiting effector T cell activation and natural killer cell function [34,35]. Further immunosuppressive effects of IDO involve reducing the immunogenicity of dendritic cells and promoting T_reg_ conversion [36]. IDO also mediates tryptophan depletion to stimulate T cell apoptosis via mTOR modulation [37,38]. Considering that immune dysregulation is one of the hallmarks of AD [14], we investigated whether IFN-γ treatment can upregulate the expression of IDO in iMSCs. We found that IFN-γ significantly increased the expression of IDO in iMSCs and observed that IFN-γ-iExo can resolve inflammation in in vivo and in vitro settings.

IFN-γ-iExo alleviated clinical scores and histology findings through EP as well as SC administration in an Af-induced AD mouse model, and the therapeutic effect was shown in a dose-dependent manner. In vitro studies using human keratinocytes showed that IFN-γ-iExo decreased the levels of innate cytokines such as TSLP, IL-25, and IL-33, which induce Th2 immune response. Additionally, IFN-γ-iExo treatment led to increases in the expression of keratinocyte differentiation markers KRT1 and KRT10 as well as the expression of CerS3 and DSG1, which are related to skin barrier function. Bioinformatics analysis using skin mRNA of the AD mouse model showed similar results to the in vitro study, in that IFN-γ-iExo treatment was primarily related to skin barrier function and multiple T cell immune response pathways. Therefore, the effect of IFN-γ-iExo on the treatment of AD may be mediated by the restoration of skin barrier defects and suppression of immune responses. Skin barrier restoration may be secondary to immunosuppression; nevertheless, considering the regenerative and anti-inflammatory effects of stem cells that are known so far, it is reasonable to infer that IFN-γ-iExo has effects on both.

From a clinical perspective, iMSC-derived exosomes hold several advantages over the direct use of their parental cells. Although the efficacy of using MSCs for alleviating AD has been reported [14], intravenously administered MSCs tend to accumulate in vascularized organs such as the lung or the liver [39,40]. Furthermore, allogenic MSCs might become tumorigenic in vivo because of chromosome alteration [41]. The use of exosomes can preclude such risks and would be especially more favorable for skin diseases considering that exosomes can be topically administered. Administration of highly concentrated IFN-γ-iExo in AD mice did not result in observable hypersensitivity reactions, while the overall clinical and histologic findings were comparable to those obtained from dexamethasone-treated mice. In addition, iMSC-derived exosomes can be produced from homologous cells from a single clone, thus providing an ideal source of exosome production. Lastly, iMSC-derived exosomes can be stored and quality-controlled more easily than parental cells.

## 4. Materials and Methods

### 4.1. Culture of IFN-γ-Primed iMSCs

iMSCs were prepared as described in our previous study [21,28]. iMSCs (passage 4) were cultured in high-glucose DMEM (HyClone, Chicago, IL, USA) with 15% FBS and 1% antibiotic antimycotic (Thermo Fisher Scientific, Waltham, MA, USA) in a T-75 flask (Eppendorf, Hamburg, Germany) at 37 °C in 5% CO_2_ and 95% humidified air. Upon reaching 90% confluency, the cells were detached using TryPLE Express (Thermo Fisher Scientific, Waltham, MA, USA) and seeded at a density of 10,000 cells/cm^2^ to a 4-layer Cell Factory system (Thermo Fisher Scientific, Waltham, MA, USA). The next day, the cells were treated with 20 ng/mL of human recombinant IFN-γ protein for 24 h (R&D Systems, Minneapolis, MI, USA), after which the media was aspirated and washed with DPBS (HyClone).

### 4.2. Isolation of Exosomes from IFN-γ-Primed iMSCs (IFN-γ-iExo)

The culture media of IFN-γ-primed iMSCs were replaced with phenol red-free DMEM supplemented with 15% EV-depleted FBS as described in our previous study [21,28]. After 3 days of incubation, the media was harvested and centrifuged for 10 min at 300× *g*, and the supernatant was centrifuged for 20 min at 2000× *g*. The supernatant was centrifuged for an additional 80 min at 10,000× *g*, and the resulting supernatant was filtered through a 0.2 μm vacuum filter (Merck Millipore, Burlington, MA, USA). Finally, exosomes were isolated by ultracentrifugation at 100,000× *g* for 80 min, and the pellet was subsequently washed with PBS and subjected to ultracentrifugation (Beckman Coulter, Brea, CA, USA). The exosome pellet was then resuspended in PBS.

### 4.3. Characterization of Exosomes

Exosome morphology was analyzed by cryo-transmission electron microscopy (cryo-TEM). Grids (Quantifoil, R2/2, 200mesh, MiTeGen, New York, NY, USA) were made hydrophilic with the glow discharge system (PELCO easiGlow™, Ted Pella, Redding, CA, USA), and 4 µL of samples were added to the grid and blotted for 1.5 s with 100% humidity at 4 °C. The samples underwent plunge-freezing in liquid ethane for vitrification with Vitrobot Mark IV (Thermo Fisher Scientific, Waltham, MA, USA) and were analyzed using a Talos L120C (TEM microscope, Thermo Fisher Scientific, Waltham, MA, USA) at 120 kV at the Nanobioimaging Center of Seoul National University (Seoul, South Korea). Nanoparticle analysis was conducted to determine the size and concentration of the exosomes using NanoSight 300 (Malvern Instruments, Malvern, UK).

### 4.4. Immunofluorescence of Indoleamine 2,3-dioxygenase (IDO)

After IFN-γ treatment, 4% paraformaldehyde was added, and cells were treated with 0.1% Triton X-100 for 10 min for permeabilization. IFN-γ-induced iMSCs were first labeled with 10 µg/mL of anti-indoleamine 2,3-dioxygenase antibody (Abcam, Cambridge, UK) for 1 h at 4 °C. After washing, the cells were labeled with Alexa Fluor 488-conjugated anti-rabbit IgG (dilution 1:1000) (Abcam), and the nuclei were stained with 4,6-diamidino-2-phenylindole (DAPI). Images were obtained using a Zeiss LSM 780 confocal microscopy system (Carl Zeiss Meditec AG, Jena, Germany).

### 4.5. Fluorescence-Activated Cell Sorting (FACS) Analysis

Phycoerythrin (PE)-conjugated mouse anti-human CD73, fluorescein isothiocyanate (FITC)-conjugated mouse anti-human CD90, PE-conjugated mouse anti-human CD105, and FITC-conjugated mouse anti-human CD34 antibodies were purchased from eBioscience (Waltham, MA, USA). MACS Plex Exosome Kit (Miltenyi Biotec, Bergisch Gladbach, Germany) was used to evaluate the exosome surface markers (CD63 and CD81). PE-conjugated mouse anti-human IDO was treated after permeabilization with 0.1% Triton X-100 in PBS for 10 min. All data were measured with the Acoustic Focusing Cytometer (Thermo Fisher Scientific, Waltham, MA, USA).

### 4.6. Immunoblotting

Exosome and cellular proteins were obtained by fractionation of cell lysates with 10% sodium dodecyl sulfate-polyacrylamide gel electrophoresis (SDS-PAGE) under reducing conditions. The protein bands were transferred onto a polyvinylidene fluoride membrane (PVDF) and incubated overnight at 4 °C with the following antibodies: rabbit monoclonal anti-CD9 (Abcam, Cambridge, UK), anti-CD63 (Abcam), anti-calnexin (Abcam), anti-CD81 (Invitrogen, MA, USA), anti-TSG101 (Invitrogen), and anti-GM130 (Cell Signaling, MA, USA). Before probing, nonspecific binding was blocked by incubation with 5% skim milk in TBST (10 mM Tris, pH 8.0, 150 mM NaCl, and 0.5% Tween-20) for 60 min at room temperature. Membranes were washed four times for 10 min each and incubated with horseradish peroxidase-linked goat anti-rabbit secondary antibody (1:3000; Abcam) at room temperature for 1 h. Blots were washed four times with TBST and developed with the enhanced chemiluminescence system (Amersham Biosciences, Waltham, MA, USA) according to the manufacturer’s protocols and were visualized using the Chemidoc imaging system (BioRad, Hercules, CA, USA).

### 4.7. Quantitative Real-Time PCR in iMSC and Human Keratinocyte HaCaT Cells

The levels of IDO mRNA were measured in IFN-γ primed iMSCs. HaCa T cells were co-treated with 50 ng/mL IL-4 and IL-13 for 24 h and then incubated with IFN-γ-iExo for 24 h. The mRNA levels of thymic stromal lymphopoietin (TSLP), IL-25, IL-33, keratin 1 (KRT1), keratin 10 (KRT10), desmoglein 1 (DSG1), and ceramide synthase 3 (CerS3) were measured in HaCa T cells. Total RNA extraction was performed with the TRIzol reagent for gene expression analysis, and cDNA was synthesized using AccuPower CycleScript RT PreMix (Bioneer, Daejeon, Korea). RT-PCR was performed using the QuantStudio 5 Flex Real-Time PCR System (Thermo Fisher Scientific, Waltham, MA, USA). The sequences of primers used were as follows: GAPDH, 5′-ACATCGCTCAGACACCATG-3′ and 5′-TGTAGTTGAGGTCAATGAAG-3′; IDO, 5′-GCCCTTCAAGTGTTTCACCAA-3′ and 5′-GCCTTTCCAGCCAGACAAATAT-3′; TSLP, 5′-TTCCTGTGGACTGGCAATGAG-3′ and 5′-ACCCAATTCCACCCCAGTTT C-3′; IL-25, 5′-GAGCGACCCAGATTAGGTGAG-3′ and 5′-TGGGTTCCCATGA CCATTGC-3′; IL-33, 5′-TGTCACATTGGCAAAGTT-3′ and 5′-CAGTAAGCAGT GTTATCAGGAA-3′; KRT1, 5′-CAACCAGAGCCTTCTTCAGC-3′ and 5′-AGGAG GCAAATTGGTTGTTG-3′; KRT10, 5′-CCTGGCTTCCTACTTGGACA-3′ and 5′-TTGCCATGCTTTTCATACCA-3′; DSG1, 5′-TCCCATTTTTGATGATCTGTTGTG-3′ and 5′-GCTCAAAGCCAGCTGCACTA-3′; CerS3, 5′ ACATTCCACAAGGCAACC ATTG-3′ and 5′-CTCTTGATTCCGCCGACTCC-3′. The changes in mRNA expression were determined according to the 2^−ΔΔCT^ method.

### 4.8. Mouse Model of AD

Female BALB/c mice (8 weeks old) were purchased from Orient Bio Inc. (Seongnam, South Korea). All experiments were designed and reported in accordance with the Animal Research: Reporting of In Vivo Experiments (ARRIVE) guidelines. Age-matched littermate mice were used in all the experiments. Same female litter mates were housed together in individually ventilated cages with 4–6 mice per cage. Each mouse group was randomly assigned a cage location. All mice were maintained on a regular 12 h light/12 h dark diurnal lighting cycle with ad libitum access to food and water. Af antigen was purchased from Greer (Lenoir, NC, USA). To induce AD-like skin, 40 µg of Af extract was epicutaneously applied daily to a 1-cm^2^ area on the shaved dorsal surface for five consecutive days (days 1–5). This procedure was repeated after a 13-day interval. In the control group (*n* = 4), PBS was applied instead of Af (*n* = 6). Mice were subcutaneously (*n* = 5) administered IFN-γ-iExo (25 µg) and epicutaneously administered IFN-γ-iExo 25 µg (*n* = 6) on day 22 and sacrificed on day 27.

To examine the dose-response effect of IFN-γ-iExo on AD, we also generated a severe AD mouse model [42] by epicutaneously applying Af extract (40 µg) to the dorsal skin of BALB/c mice for 5 consecutive days per week for 5 weeks. In the control group (*n* = 3), PBS was applied instead of Af (*n* = 6). Mice were epicutaneously administered 2.5 µg (*n* = 5) or 25 µg (*n* = 5) of IFN-γ-iExo on day 33 and sacrificed on day 38.

### 4.9. Clinical Scores and Transepidermal Water Loss (TEWL)

A single investigator assessed the clinical scores of the skin lesions of mice at the end of each experimental day. Dryness, scaling, erosion, excoriation, and hemorrhage were scored as 0 (absent), 1 (mild), 2 (moderate), and 3 (severe), and the sums of these items were used as the overall clinical score (maximum score, 15). Epidermal permeability barrier function was evaluated by measuring TEWL with a Vapometer^®^SWL-3 (Delfin Technologies Ltd., Kuopio, Finland).

### 4.10. ELISA Assay for Serum Total IgE

Mice were euthanized by CO_2_ inhalation on Day 27 or 37, and whole blood (approximately 0.5 mL) was collected from the heart. The serum was separated using a serum-separating tube and centrifugation at 2000× *g* rpm for 5 min at 20 °C. Total serum IgE concentrations were measured using the BD OptEIA ELISA set (BD Biosciences, Los Angeles, CA, USA) according to the manufacturer’s instructions.

### 4.11. Histological Examination of Mouse Skin

Samples from dorsal skin lesions were fixed with 10% formalin, embedded in paraffin, cut into 5 μm thick sections, and stained with hematoxylin-eosin and toluidine blue. The mean numbers of eosinophils, neutrophils, lymphocytes, and mast cells were calculated in eight random fields per slide (magnification, 400×). Epidermal and dermal thickness was measured using the Qupath software.

### 4.12. Gene Expression Analysis of Mouse Skin

Total RNA concentration was calculated by Quant-IT RiboGreen (#R11490; Invitrogen). A library was independently prepared with 1 μg of total RNA for IFN-γ-iExo treatment (*n* = 3) and AD (*n* = 3) mice skin using the Illumina TruSeq Stranded mRNA Sample Prep Kit (#RS-122-2101; Illumina, Inc., San Diego, CA, USA). The cleaved RNA fragments were copied into the first-strand cDNA using SuperScript II reverse transcriptase (#18064014; Invitrogen) and random primers. The products were then purified and enriched with PCR to create the final cDNA library. The libraries were quantified using the KAPA Library Quantification Kits for Illumina sequencing platforms according to the qPCR Quantification Protocol Guide (#KK4854; KAPA BIOSYSTEMS) and qualified using the TapeStation D1000 ScreenTape (#5067-5582; Agilent Technologies). Fifteen mRNA-seq samples were preprocessed and aligned to the mouse genome using Tophat [43]. The quantified gene expressions are presented in units of FPKM (fragments per kilobase of transcript per million). DEGs were identified using DEseq2 based on the gene expression count data. The DEGs were subject to pathway enrichment analysis using g:Profiler [43]. The pathways from the Reactome [44] and KEGG [45] pathways were used. The significance of pathway enrichment was tested using the g:SCS method, which corrected the *p*-value during multiple testing considering the unevenly distributed structure of the functionally annotated genes. The *p*-value correction of g:SCS was more robust than the Bonferroni method or the FDR method.

### 4.13. Statistical Analysis

Statistical analyses were performed using SPSS (Version 19, IBM Corp., Armonk, NY, USA) except for gene expression analysis of the mouse skin. For comparisons between two groups, Student’s *t*-test was used. Comparisons between multiple treatment groups and a control group were performed using one-way analysis of variance (ANOVA). For post-hoc analysis, Tukey’s HSD test was conducted. *p*-values < 0.05 were considered to be statistically significant.

## 5. Conclusions

Topical treatment with IFN-γ-iExo significantly improved both clinical and histological findings of AD in an Af-induced mouse model, likely via skin barrier restoration and suppression of T cell-mediated immune responses. Exosomes derived from the IFN-γ primed iMSCs may be considered a therapeutic option to reduce skin inflammation and restore the skin barrier function in AD.

## Data Availability

All data generated during this study are included in this article and Supplementary Files. Data are available upon reasonable request from the authors.

## Supplementary Materials

The following supporting information can be downloaded at: https://www.mdpi.com/article/10.3390/ijms241411635/s1.

## Abbreviations

AD: Atopic dermatitis; Af: *Aspergillus fumigatus;* DEG: Differentially expressed gene; FACS: Fluorescence-activated cell sorting; FITC: Fluorescein isothiocyanate; IDO: Indoleamine 2,3-dioxygenase; IFN-γ: Interferon-gamma; IgE: Immunoglobulin E; IL: Interleukin; iMSC: iPSC-derived MSC; iPSC: Induced pluripotent stem cell; MSC: Mesenchymal stem cell; PE: Phycoerythrin; PVDF: Polyvinylidene fluoride membrane; TEM: Transmission electron microscopy; TEWL: Transepidermal water loss; Th: T helper

## References

[1] Leung D.Y.M., Bieber T. (2003). Atopic dermatitis. Lancet.

[2] Bieber T. (2008). Atopic Dermatitis. N. Engl. J. Med..

[3] Boguniewicz M., Leung D.Y. (2011). Atopic dermatitis: A disease of altered skin barrier and immune dysregulation. Immunol. Rev..

[4] Brandt E.B., Sivaprasad U. (2011). Th2 Cytokines and Atopic Dermatitis. J. Clin. Cell Immunol..

[5] Astrid J., van Beelena M.B.M.T., Martien L., Kapsenberga B., de Jong E.C. (2007). Interleukin-17 in inflammatory skin disorders. Curr. Opin. Allergy Clin. Immunol..

[6] Uccelli A., Pistoia V., Moretta L. (2007). Mesenchymal stem cells: A new strategy for immunosuppression?. Trends Immunol..

[7] Seegräber M., Srour J., Walter A., Knop M., Wollenberg A. (2018). Dupilumab for treatment of atopic dermatitis. Expert Rev. Clin. Pharmacol..

[8] Nauta A.J., Fibbe W.E. (2007). Immunomodulatory properties of mesenchymal stromal cells. Blood.

[9] Uccelli A., Moretta L., Pistoia V. (2008). Mesenchymal stem cells in health and disease. Nat. Rev. Immunol..

[10] Krampera M., Cosmi L., Angeli R., Pasini A., Liotta F., Andreini A., Santarlasci V., Mazzinghi B., Pizzolo G., Vinante F. (2006). Role for interferon-gamma in the immunomodulatory activity of human bone marrow mesenchymal stem cells. Stem Cells.

[11] Li N., Hua J. (2017). Interactions between mesenchymal stem cells and the immune system. Cell Mol. Life Sci..

[12] Kim H.S., Yun J.W., Shin T.H., Lee S.H., Lee B.C., Yu K.R., Seo Y., Lee S., Kang T.W., Choi S.W. (2015). Human umbilical cord blood mesenchymal stem cell-derived PGE2 and TGF-beta1 alleviate atopic dermatitis by reducing mast cell degranulation. Stem Cells.

[13] Kim H.S., Lee J.H., Roh K.H., Jun H.J., Kang K.S., Kim T.Y. (2017). Clinical Trial of Human Umbilical Cord Blood-Derived Stem Cells for the Treatment of Moderate-to-Severe Atopic Dermatitis: Phase I/IIa Studies. Stem Cells.

[14] Park A., Park H., Yoon J., Kang D., Kang M.H., Park Y.Y., Suh N., Yu J. (2019). Priming with Toll-like receptor 3 agonist or interferon-gamma enhances the therapeutic effects of human mesenchymal stem cells in a murine model of atopic dermatitis. Stem Cell Res. Ther..

[15] Phinney D.G., Pittenger M.F. (2017). Concise Review: MSC-Derived Exosomes for Cell-Free Therapy. Stem Cells.

[16] Brown P.T., Squire M.W., Li W.J. (2014). Characterization and evaluation of mesenchymal stem cells derived from human embryonic stem cells and bone marrow. Cell Tissue Res..

[17] Zhao C., Ikeya M. (2018). Generation and Applications of Induced Pluripotent Stem Cell-Derived Mesenchymal Stem Cells. Stem Cells Int..

[18] Samsonraj R.M., Raghunath M., Nurcombe V., Hui J.H., van Wijnen A.J., Cool S.M. (2017). Concise Review: Multifaceted Characterization of Human Mesenchymal Stem Cells for Use in Regenerative Medicine. Stem Cells Transl. Med..

[19] Maas S.L.N., Breakefield X.O., Weaver A.M. (2017). Extracellular Vesicles: Unique Intercellular Delivery Vehicles. Trends Cell Biol..

[20] Riazifar M., Pone E.J., Lotvall J., Zhao W. (2017). Stem Cell Extracellular Vesicles: Extended Messages of Regeneration. Annu. Rev. Pharmacol. Toxicol..

[21] Kim J., Lee S.K., Jeong S.Y., Cho H.J., Park J., Kim T.M., Kim S. (2021). Cargo proteins in extracellular vesicles: Potential for novel therapeutics in non-alcoholic steatohepatitis. J. Nanobiotechnol..

[22] Collino F., Pomatto M., Bruno S., Lindoso R.S., Tapparo M., Sicheng W., Quesenberry P., Camussi G. (2017). Exosome and Microvesicle-Enriched Fractions Isolated from Mesenchymal Stem Cells by Gradient Separation Showed Different Molecular Signatures and Functions on Renal Tubular Epithelial Cells. Stem Cell Rev. Rep..

[23] Toh W.S., Lai R.C., Zhang B., Lim S.K. (2018). MSC exosome works through a protein-based mechanism of action. Biochem. Soc. Trans..

[24] Bjorge I.M., Kim S.Y., Mano J.F., Kalionis B., Chrzanowski W. (2017). Extracellular vesicles, exosomes and shedding vesicles in regenerative medicine—A new paradigm for tissue repair. Biomater. Sci..

[25] Spees J.L., Lee R.H., Gregory C.A. (2016). Mechanisms of mesenchymal stem/stromal cell function. Stem Cell Res. Ther..

[26] Tamura R., Uemoto S., Tabata Y. (2016). Immunosuppressive effect of mesenchymal stem cell-derived exosomes on a concanavalin A-induced liver injury model. Inflamm. Regen..

[27] Ren G., Su J., Zhang L., Zhao X., Ling W., L’Huillie A., Zhang J., Lu Y., Roberts A.I., Ji W. (2009). Species variation in the mechanisms of mesenchymal stem cell-mediated immunosuppression. Stem Cells.

[28] Kim S., Lee S.K., Kim H., Kim T.M. (2018). Exosomes Secreted from Induced Pluripotent Stem Cell-Derived Mesenchymal Stem Cells Accelerate Skin Cell Proliferation. Int. J. Mol. Sci..

[29] Sheng H., Wang Y., Jin Y., Zhang Q., Zhang Y., Wang L., Shen B., Yin S., Liu W., Cui L. (2008). A critical role of IFNgamma in priming MSC-mediated suppression of T cell proliferation through up-regulation of B7-H1. Cell Res..

[30] Meisel R., Zibert A., Laryea M., Gobel U., Daubener W., Dilloo D. (2004). Human bone marrow stromal cells inhibit allogeneic T-cell responses by indoleamine 2,3-dioxygenase-mediated tryptophan degradation. Blood.

[31] Polchert D., Sobinsky J., Douglas G., Kidd M., Moadsiri A., Reina E., Genrich K., Mehrotra S., Setty S., Smith B. (2008). IFN-gamma activation of mesenchymal stem cells for treatment and prevention of graft versus host disease. Eur. J. Immunol..

[32] Hoogduijn M.J., Popp F., Verbeek R., Masoodi M., Nicolaou A., Baan C., Dahlke M.H. (2010). The immunomodulatory properties of mesenchymal stem cells and their use for immunotherapy. Int. Immunopharmacol..

[33] Holmgaard R.B., Zamarin D., Li Y., Gasmi B., Munn D.H., Allison J.P., Merghoub T., Wolchok J.D. (2015). Tumor-Expressed IDO Recruits and Activates MDSCs in a Treg-Dependent Manner. Cell Rep..

[34] Pietra G., Vitale M., Moretta L., Mingari M.C. (2012). How melanoma cells inactivate NK cells. Oncoimmunology.

[35] Wang D., Saga Y., Mizukami H., Sato N., Nonaka H., Fujiwara H., Takei Y., Machida S., Takikawa O., Ozawa K. (2012). Indoleamine-2,3-dioxygenase, an immunosuppressive enzyme that inhibits natural killer cell function, as a useful target for ovarian cancer therapy. Int. J. Oncol..

[36] Nguyen N.T., Kimura A., Nakahama T., Chinen I., Masuda K., Nohara K., Fujii-Kuriyama Y., Kishimoto T. (2010). Aryl hydrocarbon receptor negatively regulates dendritic cell immunogenicity via a kynurenine-dependent mechanism. Proc. Natl. Acad. Sci. USA.

[37] Zhai L., Spranger S., Binder D.C., Gritsina G., Lauing K.L., Giles F.J., Wainwright D.A. (2015). Molecular Pathways: Targeting IDO1 and Other Tryptophan Dioxygenases for Cancer Immunotherapy. Clin. Cancer Res..

[38] Wu H., Gong J., Liu Y. (2018). Indoleamine 2, 3-dioxygenase regulation of immune response (Review). Mol. Med. Rep..

[39] Karp J.M., Leng Teo G.S. (2009). Mesenchymal stem cell homing: The devil is in the details. Cell Stem Cell.

[40] Leibacher J., Henschler R. (2016). Biodistribution, migration and homing of systemically applied mesenchymal stem/stromal cells. Stem Cell Res. Ther..

[41] Baldari S., Di Rocco G., Piccoli M., Pozzobon M., Muraca M., Toietta G. (2017). Challenges and Strategies for Improving the Regenerative Effects of Mesenchymal Stromal Cell-Based Therapies. Int. J. Mol. Sci..

[42] Park A., Park H., Yu J. (2019). Development of Aspergillus fumigatus-induced chronic atopic dermatitis mouse model. Allergy Asthma Respir. Dis..

[43] Raudvere U., Kolberg L., Kuzmin I., Arak T., Adler P., Peterson H., Vilo J. (2019). g:Profiler: A web server for functional enrichment analysis and conversions of gene lists (2019 update). Nucleic Acid. Res..

[44] Jassal B., Matthews L., Viteri G., Gong C., Lorente P., Fabregat A., Sidiropoulos K., Cook J., Gillespie M., Haw R. (2020). The reactome pathway knowledgebase. Nucleic Acids Res..

[45] Kanehisa M., Furumichi M., Sato Y., Ishiguro-Watanabe M., Tanabe M. (2021). KEGG: Integrating viruses and cellular organisms. Nucleic Acids Res..

